# Supplementary material for: Optimized Properties in Multifunctional Polyphenylene Sulfide Composites via Graphene Nanosheets/Boron Nitride Nanosheets Dual Segregated Structure under High Pressure
Source: Nanomaterials (Basel). 2022 Oct 10;12(19):3543. doi: 10.3390/nano12193543 (PMC9565237; doi:10.3390/nano12193543)
Supplement: Supplementary file 1 [file nanomaterials-12-03543-s001.zip › nanomaterials-1915489-supplementary.pdf]

nanomaterials

Supplementary Materials

Optimized Properties in Multifunctional Polyphenylene Sulfide Composites via Graphene Nanosheets/Boron Nitride Nanosheets Dual Segregated Structure under High Pressure

Liangqing Zhang [1], Shugui Yang [2], Longgui Peng [1], Kepeng Zhong [3] and Yanhui Chen [3,*]

[1] College of Material Science and Engineering, Xi'an University of Science and Technology, Xi'an 710054, China
[2] Shaanxi International Research Center for Soft Matter, State Key Laboratory for Mechanical Behavior of Materials, Xi'an Jiaotong University, Xi'an 710049, China
[3] School of Chemistry and Chemical Engineering, Shaanxi Key Laboratory of Macromolecular Science and Technology, Key Laboratory of Special Functional and Smart Polymer Materials, Ministry of Industry and Information Technology, Northwestern Polytechnical University, Xi'an 710072, China
* Correspondence: yanhuichen@nwpu.edu.cn; Tel.: +86-029-88431656

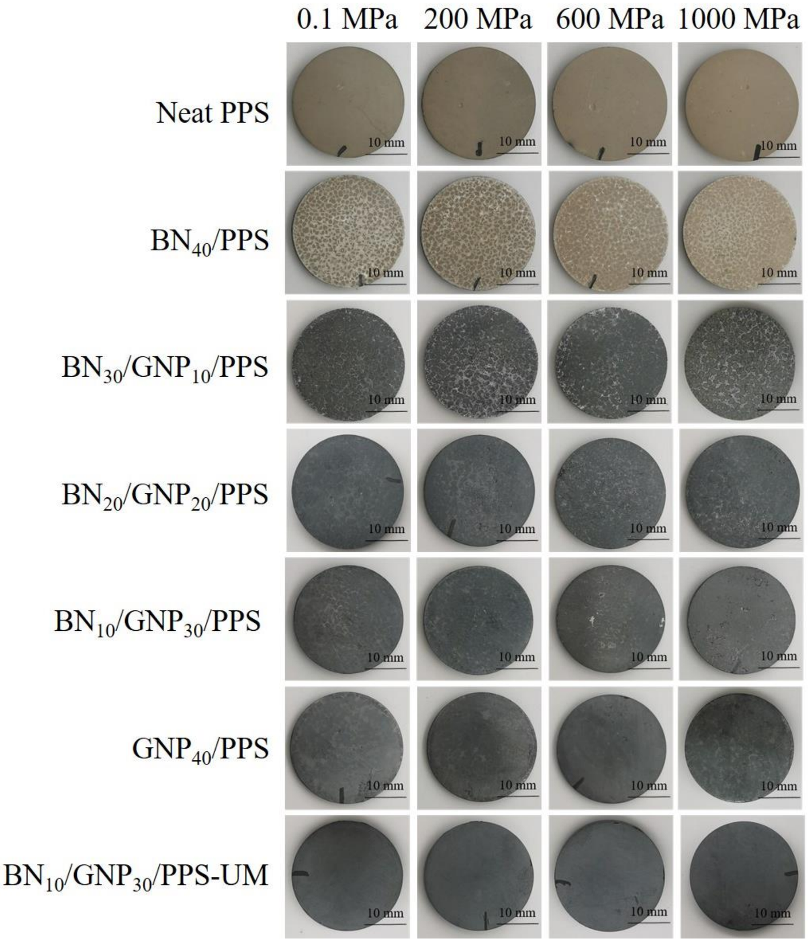

Figure S1. Digital images of cylindrical tablets of PPS composites hot pressed under different pressures.

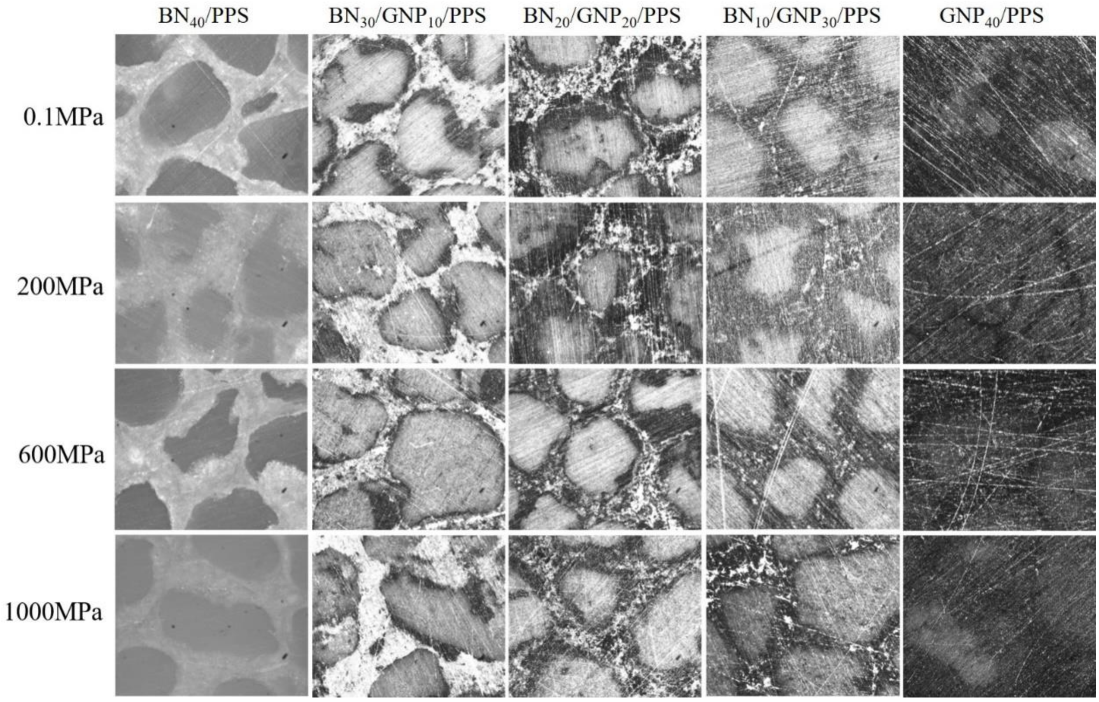

Figure S2. OM images of PPS composites processed under different pressures.

Table S1. Integrated comparation of electrical conductivity and EMI SE among BN/GNP/PPS composites and other previous reported polymer composites with segregated structure.

Composites	Filler content	Conductivity (S/m)	Thickness (mm)	EMI SE (dB)	Ref.
CNT/PLA	1.0 wt%	12	2.7	35.5	1
MWCNT/PS	7.0 wt%	61.9	1.8	42	2
Ni/UHWMPE	2.58 vol%	2648	2	55	3
EG/PA6	2.27 vol%	0.55	2	25	4
MWCNTs/epoxy	4 wt%	6.8	2	31.5	5
MWCNT/PS/SiAPP	7 wt%	--	3	11	6
MWCNT/PEBA	1.12 vol%	3.18E-5	1.6	16	7
PP/APP/CNT	7 wt%	37.8	2	32.1	8
CNT/PE	5 wt%	15	2.1	46.4	9
G-CNT/UHMWPE	15 wt%	195.3	2.1	81	10
MWCNT/PMMA	5 wt%	3.19	2	35.9	11
rGO/PMMA	2.6 vol%	91.2	2	63.2	12
Ag/CNT/PLLA/PCL	2.44 vol%	70	2	43.3	13
MWCNT/PLA	0.0054 vol%	6.3	5	45	14
MWCNT/PVDF	7 wt%	6	3	30.89	15
CNT/GO/PU	10 wt%	52	2	52.7	16
CNT/PU	7 wt%	20	2	41.2	17
CNT/PP	5.0 wt%	117	2.2	48.3	18
rGO/PS	3.47 vol%	43.5	2.5	45.1	19
rGO/UHMWPE	0.66 vol%	3.4	2.5	32.4	20
CNT/PVDF	4 wt%	74.5	2	36.8	21
CNT/UHMWPE	2 wt%	49.7	2	33.5	22
CNT/Epoxy	1 wt%	34.5	1.9	22	23

MWCNT/PLA	0.8 vol%	30	1.5	25	[24]
Ag/PLA	5.89 vol%	254	1.5	50	[25]
CNT/PPS	5.0 wt%	72	2	49.6	[26]
rGO/PMMA	2.6 vol%	91.2	2.9	63.2	[12]
GNP/BN/PPS	30 wt%	0.07	2	70	This work

Table S2. Integrated comparation of thermal conductivity and EMI SE among BN/GNP/PPS composites and other previous reported polymer composites.

Composites	Filler content	Thermal conductivity (W/m/K)	EMI SE (dB)	Ref.
Cenospheres/ Fe_3O_4/MCMB/CNT/PAN	---	4.2	80.5	[27]
SiC/CNT/MCMB/PAN	---	6.5	67	[28]
GNP/PS	35 wt%	4.72	33	[29]
Mxene/PVA	19.5 wt%	4.57	44.4	[30]
NCCF/MWCNT/PU	---	0.032	24.7	[31]
MWCNT/POM	40 wt%	1.95	45.7	[32]
GNP/POM	48 wt%	4.24	44.7	[32]
Graphene/PDMS	18.1 wt%	3	86	[33]
Ag/CF/epoxy	7 wt%	2.49	38	[34]
GNP/rGO/epoxy	20.5 wt%	1.56	51	[35]
Ag/cellulose	50 wt%	10.55	101	[36]
Graphene/SiC/PVDF	9.5 wt%	2.13	32.5	[37]
Graphen/Ni/PVDF	20 wt%	8.96	43.3	[38]
Ag/TPU	15 vol%	4.45	105.3	[39]
MXene/BN/PDMS	---	0.65	35.2	[40]
MXene/PAT/PANI–PpAP	---	0.687	45.18	[41]
GNS/CINAP/CE	20 wt%	4.13	55	[42]
Carbon network/epoxy	7.0 vol%	0.58	27.8	[43]
AgNW/ Fe_3O_4/MF	---	0.034	49	[44]
CCA/rGO/PDMS	3.05 wt%	0.65	51	[45]
MWCNT/PLA	0.0054 vol%	0.03	50	[14]
MWCNT-Fe3O4@Ag	---	0.46	35	[46]
rGO-WPU/cotton	2 wt%	2.13	48.1	[47]
CNT@PDA/EVA	70 wt%	17.9	32.4	[48]
polyacrylate/graphene@polydopamine	20 wt%	1.68	58	[49]
CuNWs-TAGA/epoxy	7.2 wt%	0.51	47	[50]
CNT/acrylic	0.62 vol%	0.008	36.7	[51]
Ni/PVDF	10 wt%	0.075	26.8	[52]
MXene/AgNW/CNF	---	15.5	55.9	[53]
CL@GC/CE	---	0.61	35.57	[54]
GNP/BN/PPS	40 wt%	6.838	70	This work
Graphene Fe–Ni		4.1	55	[42]
PC/SAN/G-NI		0.7	29.4	[55]
Carbon monolith/RGO		0.057	43	[56]

MXD6/EG/CNTs	2.1	50	57
WCM/N-G/ AgNWs	0.14	60	58
Graphene/SBR matrix	2.9	45	59
Titanium foam	0.8	45	60
PVDF/GNP-Ni	0.66	57.3	61

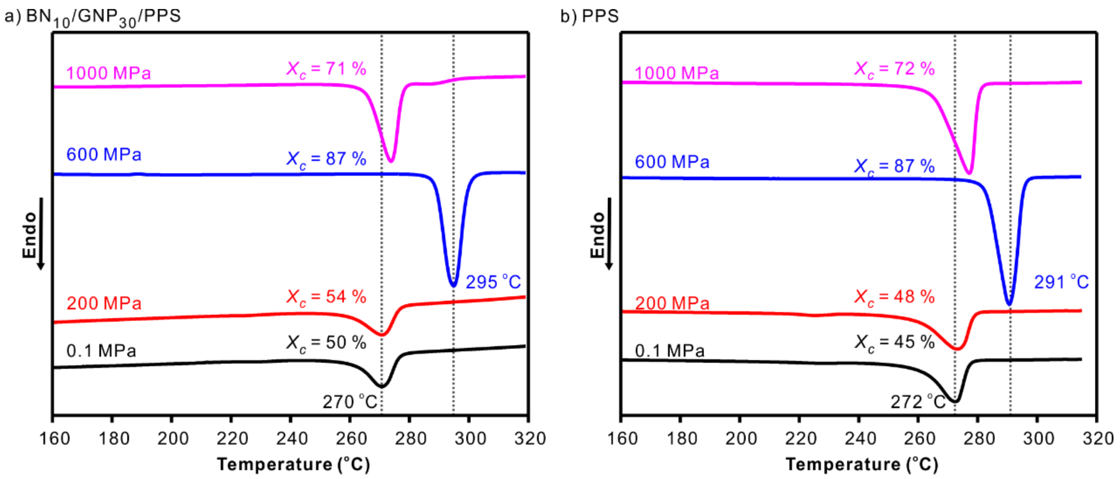

Figure S3. DSC heating curves of $BN_{10}/GNP_{30}/PPS$ with segregated structures (a) and neat PPS (b) processed under different pressures.

The DSC heating curves of $BN_{10}/GNP_{30}/PPS$ with segregated structures and neat PPS are presented in Figure S3. Obviously, both PPS composites and neat PPS display a much larger melting peak when they were crystallized under 600 MPa and 1000 MPa. The crystallinity of both PPS composites and neat PPS increases from ~50% of processed under 0.1 MPa and 200 MPa to ~80% of processed under 600 MPa and 1000 MPa, owing to the high pressuce (600 MPa and 1000 MPa) accelerates the crystallization of PPS. Additional intriguing phenomenon observed here is the PPS samples crystallized at 600 MPa show an increased melting temperature (~295 °C) comparing with that processed under other pressures (~270 °C). This is due to the formation of extended-chain crystals in PPS matrix, which would be discussed in the forthcoming paper.

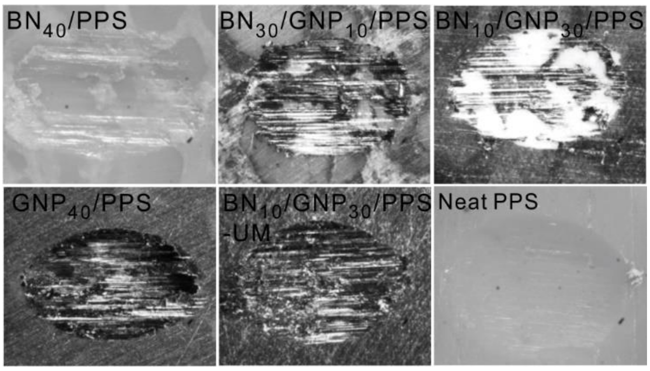

Figure S4. OM observations of worn surfaces of PPS composites and neat PPS after the test.

Figure S4 show OM images of the wear surfaces. In general, groove marks, along with surface lumps and detached areas on the wear surface are clearly visible, suggesting that the dominant wear mechanism are abrasion by surface asperities on the pin, and adhesion in the contact area.

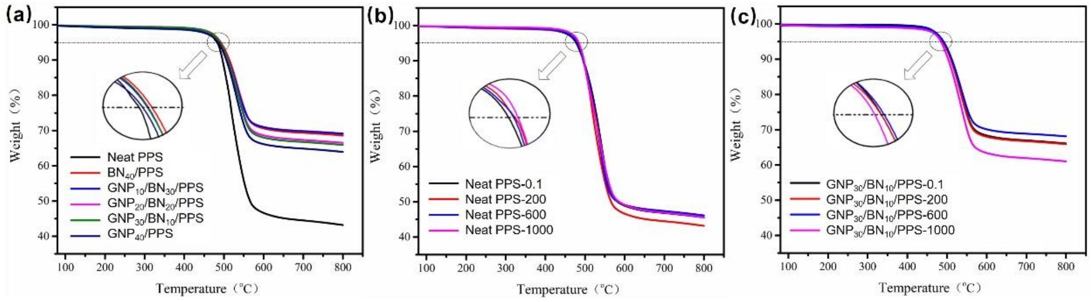

Figure S5. TGA curves of Neat PPS and GNP/BN/PPS composites at 200 MPa (a); TGA curves of Neat PPS (b) and GNP_{30}/BN_{10}/PPS composites (c) under different molding pressures.

Table S3. The compressive strength of neat PPS and PPS composites processed under different pressures (MPa).

Samples	0.1 MPa	200 MPa	600 MPa	1000 MPa
Neat PPS	163.5	165.8	163.5	164.7
BN_{40}/PPS	107.4	110.9	93.4	95.7
$GNPs_{10}/BN_{30}$/PPS	105.1	100.4	93.4	95.8
$GNPs_{20}/BN_{20}$/PPS	116.8	116.8	110.9	105.1
$GNPs_{30}/BN_{10}$/PPS	140.1	133.1	129.6	128.5
GNP_{40}/PPS	146.0	141.3	137.8	134.3
GNP_{30}/BN_{10}/PPS-UM	156.5	156.5	153.0	154.2

References

1. Cui, C.H.; Yan, D.X.; Pang, H.; Xu, X.; Jia, L.C.; Li, Z.M. Formation of a Segregated Electrically Conductive Network Structure in a Low-Melt-Viscosity Polymer for Highly Efficient Electromagnetic Interference Shielding. *ACS Sustain. Chem. Eng.* **2016,** *4*, 4137–4145.
2. Chen, J.; Liao, X.; Xiao, W.; Yang, J.M.; Jiang, Q.Y.; Li, G.X. Facile and Green Method To Structure Ultralow-Threshold and Lightweight Polystyrene/MWCNT Composites with Segregated Conductive Networks for Efficient Electromagnetic Interference Shielding. *ACS Sustain. Chem. Eng.* **2019**, *7*, 9904–9915.
3. Duan, H.J.; Xu, Y.D.; Yan, D.X.; Yang, Y.Q.; Zhao, G.Z.; Liu, Y.Q. Ultrahigh molecular weight polyethylene composites with segregated nickel conductive network for highly efficient electromagnetic interference shielding. *Mater. Lett.* **2017**, *209*, 353–356.
4. Duan, H.J.; Zhu, H.X.; Yang, Y.Q.; Hou, T.T.; Zhao, G.Z.; Liu, Y.Q. Facile and economical fabrication of conductive polyamide 6 composites with segregated expanded graphite networks for efficient electromagnetic interference shielding. *J. Mater. Sci. - Mater. Electron*. **2018,** *29*, 1058–1064.
5. Fang, H.G.; Ye, W.J.; Yang, K.J.; Song, K.; Wei, H.B.; Ding, Y.S. Vitrimer chemistry enables epoxy nanocomposites with mechanical robustness and integrated conductive segregated structure for high performance electromagnetic interference shielding. *Compos. B Eng.* **2021,** *215*, 108782.
6. Gao, C.Q.; Shi, Y.Q.; Chen, Y.J.; Zhu, S.C.; Feng, Y.Z.; Lv, Y.C.; Yang, F.Q.; Liu, M.H.; Shui, W. Constructing segregated polystyrene composites for excellent fire resistance and electromagnetic wave shielding. *J. Colloid Interface Sci*. **2022**, *606*, 1193–1204.
7. Ge, C.B.A.; Wang, G.L.; Zhao, G.Q.; Wei, C.; Li, X.Y. Lightweight and flexible poly(ether-block-amide)/multiwalled carbon nanotube composites with porous structure and segregated conductive networks for electromagnetic shielding applications. *Compos. - A: Appl. Sci. Manuf.* **2021,** *144*, 106356.
8. Hu, Y.L.; Feng, D.; Xie, Y.H.; Xie, D.L. Microwave-Assisted Confining Flame-Retardant Polypropylene in Carbon Nanotube Conductive Networks for Improved Electromagnetic Interference Shielding and Flame Retardation. *Advanced Engineering Materials* **2021,** *23*, 2100024.

9. Jia, L.C.; Yan, D.X.; Cui, C.H.; Jiang, X.; Ji, X.; Li, Z.M. Electrically conductive and electromagnetic interference shielding of polyethylene composites with devisable carbon nanotube networks. *J. Mater. Chem. C* **2015,** *3*, 9369–9378.
10. Jia, L.C.; Yan, D.X.; Jiang, X.; Pang, H.; Gao, J.F.; Ren, P.G.; Li, Z.M. Synergistic Effect of Graphite and Carbon Nanotubes on Improved Electromagnetic Interference Shielding Performance in Segregated Composites. *Ind. Eng. Chem. Res.* **2018,** *57*, 11929–11938.
11. Li, T.; Zhao, G.; Zhang, L.; Wang, G.; Li, B.; Gong, J. Ultralow-threshold and efficient EMI shielding PMMA/MWCNTs composite foams with segregated conductive network and gradient cells. *Express Polym. Lett.* **2020,** *14* , 685–703.
12. Sharif, F.; Arjmand, M.; Moud, A.A.; Sundararaj, U.; Roberts, E.P.L. Segregated Hybrid Poly(methyl methacrylate)/Graphene/Magnetite Nanocomposites for Electromagnetic Interference Shielding. *ACS Appl. Mater. Interfaces* **2017,** *9*, 14171–14179.
13. Tang, X.H.; Tang, Y.; Wang, Y.; Weng, Y.X.; Wang, M. Interfacial metallization in segregated poly (lactic acid)/poly (e-caprolactone)/multi-walled carbon nanotubes composites for enhancing electromagnetic interference shielding. *Compos. - A: Appl. Sci. Manuf.* **2020,** *139,* 106116.
14. Wang, G.L.; Wang, L.; Mark, L.H.; Shaayegan, V.; Wang, G.Z.; Li, H.P.; Zhao, G.Q.; Park, C.B. Ultralow-Threshold and Lightweight Biodegradable Porous PLA/MWCNT with Segregated Conductive Networks for High-Performance Thermal Insulation and Electromagnetic Interference Shielding Applications. *ACS Appl. Mater. Interfaces* **2018,** *10* , 1195–1203.
15. Wang, H.; Zheng, K.; Zhang, X.; Du, T.X.; Xiao, C.; Ding, X.; Bao, C.; Chen, L.; Tian, X.Y. Segregated poly(vinylidene fluoride)/MWCNTs composites for high-performance electromagnetic interference shielding. *Compos. - A: Appl. Sci. Manuf.* **2016,** *90*, 606–613.
16. Wang, T.; Kong, W.W.; Yu, W.C.; Gao, J.F.; Dai, K.; Yan, D.X.; Li, Z.M. A Healable and Mechanically Enhanced Composite with Segregated Conductive Network Structure for High-Efficient Electromagnetic Interference Shielding. *Nano-Micro Lett.* **2021,** *13*, 1–14.
17. Wang, T.; Yu, W.C.; Sun, W.J.; Jia, L.C.; Gao, J.F.; Tang, J.H.; Su, H.J.; Yan, D.X.; Li, Z.M. Healable polyurethane/carbon nanotube composite with segregated structure for efficient electromagnetic interference shielding. *Compos. Sci. Technol.* **2020,** *200,* 108446.
18. Wu, H.Y.; Jia, L.C.; Yan, D.X.; Gao, J.F.; Zhang, X.P.; Ren, P.G.; Li, Z.M. Simultaneously improved electromagnetic interference shielding and mechanical performance of segregated carbon nanotube/polypropylene composite via solid phase molding. *Compos. Sci. Technol.* **2018**, *156*, 87–94.
19. Yan, D.X.; Pang, H.; Li, B.; Vajtai, R.; Xu, L.; Ren, P.G.; Wang, J.H.; Li, Z.M. Structured Reduced Graphene Oxide/Polymer Composites for Ultra-Efficient Electromagnetic Interference Shielding. *Adv. Funct. Mater*. **2015,** *25*, 559–566.
20. Yan, D.X.; Pang, H.; Xu, L.; Bao, Y.; Ren, P.G.; Lei, J.; Li, Z.M. Electromagnetic interference shielding of segregated polymer composite with an ultralow loading of in situ thermally reduced graphene oxide. *Nanotechnology* **2014,** *25*, 145705.
21. Yu, W.C.; Wang, T.; Zhang, G.Q.; Wang, Z.G.; Yin, H.M.; Yan, D.X.; Xu, J.Z.; Li, Z.M. Largely enhanced mechanical property of segregated carbon nanotube/poly (vinylidene fluoride) composites with high electromagnetic interference shielding performance. *Compos. Sci. Technol.* **2018**, *167*, 260–267.
22. Yu, W.C.; Zhang, G.Q.; Liu, Y.H.; Xu, L.; Yan, D.X.; Huang, H.D.; Tang, J.H.; Xu, J.Z.; Li, Z.M. Selective electromagnetic interference shielding performance and superior mechanical strength of conductive polymer composites with oriented segregated conductive networks. *Chem. Eng. J.* **2019**, *373*, 556–564.
23. Yuan, D.; Guo, H.C.; Ke, K.; Manas-Zloczower, I. Recyclable conductive epoxy composites with segregated filler network structure for EMI shielding and strain sensing. *Compos. - A: Appl. Sci. Manuf.* **2020,** *132*, 105837.
24. Zhang, K.; Li, G.H.; Feng, L.M.; Wang, N.; Guo, J.; Sun, K.; Yu, K.X.; Zeng, J.B.; Li, T.X.; Guo, Z.H.; et al. Ultralow percolation threshold and enhanced electromagnetic interference shielding in poly(L-lactide)/multi-walled carbon nanotube nanocomposites with electrically conductive segregated networks. *J. Mater. Chem. C* **2017,** *5*, 9359–9369.
25. Zhang, K.; Yu, H.O.; Yu, K.X.; Gao, Y.; Wang, M.; Li, J.; Guo, S.Y. A facile approach to constructing efficiently segregated conductive networks in poly(lactic acid)/silver nanocomposites via silver plating on microfibers for electromagnetic interference shielding. *Compos. Sci. Technol.* **2018**, *156*, 136–143.
26. Zhang, X.P.; Jia, L.C.; Zhang, G.; Yan, D.X.; Li, Z.M. A highly efficient and heat- resistant electromagnetic interference shielding carbon nanotube/poly(phenylene sulfide) composite via sinter molding. *J. Mater. Chem. C* **2018,** *6*, 10760–10766.
27. Chaudhary, A.; Kumar, R.; Dhakate, S.R.; Kumari, S. Scalable development of a multi-phase thermal management system with superior EMI shielding properties. *Compos. B Eng.* **2019**, *158*, 206–217.
28. Chaudhary, A.; Teotia, S.; Kumar, R.; Gupta, V.; Dhakate, S.R.; Kumari, S., Multi-component framework derived SiC composite paper to support efficient thermal transport and high EMI shielding performance. *Compos. B Eng.* **2019,** *176*, 107123.
29. Guo, Y.Q.; Pan, L.L.; Yang, X.T.; Ruan, K.P.; Han, Y.X.; Kong, J.; Gu, J.W. Simultaneous improvement of thermal conductivities and electromagnetic interference shielding performances in polystyrene composites via constructing interconnection oriented networks based on electrospinning technology. *Compos. - A: Appl. Sci. Manuf.* **2019,** *124,* 105484.
30. Jin, X.X.; Wang, J.F.; Dai, L.Z.; Liu, X.Y.; Li, L.; Yang, Y.Y.; Cao, Y.X.; Wang, W.J.; Wu, H.; Guo, S.Y. Flame-retardant poly(vinyl alcohol)/MXene multilayered films with outstanding electromagnetic interference shielding and thermal conductive performances. *Chem. Eng. J.* **2020,** *380,* 122475.
31. Kim, J.M.; Lee, Y.; Jang, M.G.; Han, C.; Kim, W.N. Electrical conductivity and EMI shielding effectiveness of polyurethane foam-conductive filler composites. *J. Appl. Polym. Sci.* **2017,** *134*, 44373.
32. Li, J.; Wang, Y.; Yue, T.N.; Gao, Y.N.; Shi, Y.D.; Shen, J.B.; Wu, H.; Wang, M. Robust electromagnetic interference shielding, joule

heating, thermal conductivity, and anti-dripping performances of polyoxymethylene with uniform distribution and high content of carbon-based nanofillers. *Compos. Sci. Technol.* **2021**, *206*, 108681.
33. Li, J.C.; Zhao, X.Y.; Wu, W.J.; Ji, X.W.; Lu, Y.L.; Zhang, L.Q. Bubble-templated rGO-graphene nanoplatelet foams encapsulated in silicon rubber for electromagnetic interference shielding and high thermal conductivity. *Chem. Eng. J.* **2021**, *415*, 129054.
34. Li, J.P.; Qi, S.H.; Zhang, M.Y.; Wang, Z.F. Thermal conductivity and electromagnetic shielding effectiveness of composites based on Ag-plating carbon fiber and epoxy. *J. Appl. Polym. Sci.* **2015**, *132*, 42306.
35. Liang, C.B.; Qiu, H.; Han, Y.Y.; Gu, H.B.; Song, P.; Wang, L.; Kong, J.; Cao, D.P.; Gu, J.W. Superior electromagnetic interference shielding 3D graphene nanoplatelets/reduced graphene oxide foam/epoxy nanocomposites with high thermal conductivity. *J. Mater. Chem. C* **2019**, *7*, 2725–2733.
36. Liang, C.B.; Ruan, K.P.; Zhang, Y.L.; Gu, J.W. Multifunctional Flexible Electromagnetic Interference Shielding Silver Nanowires/Cellulose Films with Excellent Thermal Management and Joule Heating Performances. *ACS Appl. Mater. Interfaces* **2020**, *12*, 18023–18031.
37. Liang, C.Y.; Hamidinejad, M.; Ma, L.; Wang, Z.J.; Park, C.B. Lightweight and flexible graphene/SiC-nanowires/poly(vinylidene fluoride) composites for electromagnetic interference shielding and thermal management. *Carbon* **2020**, *156*, 58–66.
38. Liang, L.Y.; Xu, P.H.; Wang, Y.F.; Shang, Y.; Ma, J.M.; Su, F.M.; Feng, Y.Z.; He, C.G.; Wang, Y.M.; Liu, C.T. Flexible polyvinylidene fluoride film with alternating oriented graphene/Ni nanochains for electromagnetic interference shielding and thermal management. *Chem. Eng. J.* **2020**, *395*, 125209.
39. Liu, C.; Wu, W.; Wang, Y.; Wang, Z.Y.; Chen, Q.M. Silver-coated thermoplastic polyurethane hybrid granules for dual-functional elastomer composites with exceptional thermal conductive and electromagnetic interference shielding performances. *Compos. Commun.* **2021**, *25*, 100719.
40. Liu, H.B.; Fu, R.L.; Su, X.Q.; Wu, B.Y.; Wang, H.; Xu, Y.; Liu, X.H. Electrical insulating MXene/PDMS/BN composite with enhanced thermal conductivity for electromagnetic shielding application. *Compos. Commun.* **2021**, *23*, 100593.
41. Raagulan, K.; Braveenth, R.; Kim, B.M.; Lim, K.J.; Lee, S.B.; Kim, M.; Chai, K.Y. An effective utilization of MXene and its effect on electromagnetic interference shielding: Flexible, free-standing and thermally conductive composite from MXene-PAT-poly(p-aminophenol)-polyaniline co-polymer. *RSC Adv.* **2020**, *10*, 1613–1633.
42. Ren, F.; Song, D.P.; Li, Z.; Jia, L.C.; Zhao, Y.C.; Yan, D.X.; Ren, P.G. Synergistic effect of graphene nanosheets and carbonyl iron-nickel alloy hybrid filler on electromagnetic interference shielding and thermal conductivity of cyanate ester composites. *J. Mater. Chem. C* **2018**, *6*, 1476–1486.
43. Shen, Z.M.; Feng, J.C. Preparation of Thermally Conductive Polymer Composites with Good Electromagnetic Interference Shielding Efficiency Based on Natural Wood-Derived Carbon Scaffolds. *ACS Sustain. Chem. Eng.* **2019**, *7*, 6259–6266.
44. Shi, H.G.; Zhao, H.B.; Liu, B.W.; Wang, Y.Z. Multifunctional Flame-Retardant Melamine-Based Hybrid Foam for Infrared Stealth, Thermal Insulation, and Electromagnetic Interference Shielding. *ACS Appl. Mater. Interfaces* **2021**, *13*, 26505–26514.
45. Song, P.; Liu, B.; Liang, C.B.; Ruan, K.P.; Qiu, H.; Ma, Z.L.; Guo, Y.Q.; Gu, J.W. Lightweight, Flexible Cellulose-Derived Carbon Aerogel@Reduced Graphene Oxide/PDMS Composites with Outstanding EMI Shielding Performances and Excellent Thermal Conductivities. *Nano-Micro Lett.* **2021**, *13*, 1–17.
46. Wang, L.; Qiu, H.; Liang, C.B.; Song, P.; Han, Y.X.; Han, Y.X.; Gu, J.W.; Kong, J.; Pan, D.; Guo, Z.H. Electromagnetic interference shielding MWCNT-Fe_3O_4@Ag/epoxy nanocomposites with satisfactory thermal conductivity and high thermal stability. *Carbon* **2019**, *141*, 506–514.
47. Wang, Y.; Wang, W.; Xu, R.; Zhu, M.F.; Yu, D. Flexible, durable and thermal conducting thiol-modified rGO-WPU/cotton fabric for robust electromagnetic interference shielding. *Chem. Eng. J.* **2019**, *360*, 817–828.
48. Wang, Z.G.; Yang, Y.L.; Zheng, Z.L.; Lan, R.T.; Dai, K.; Xu, L.; Huang, H.D.; Tang, J.H.; Xu, J.Z.; Li, Z.M. Achieving excellent thermally conductive and electromagnetic shielding performance by nondestructive functionalization and oriented arrangement of carbon nanotubes in composite films. *Compos. Sci. Technol.* **2020**, *194*, 108190.
49. Wei, L.F.; Ma, J.Z.; Zhang, W.B.; Bai, S.L.; Ren, Y.N.; Zhang, L.; Wu, Y.K.; Qin, J.B. pH triggered hydrogen bonding for preparing mechanically strong, electromagnetic interference shielding and thermally conductive waterborne polymer/graphene@polydopamine composites. *Carbon* **2021**, *181*, 212–224.
50. Yang, X.T.; Fan, S.G.; Li, Y.; Guo, Y.Q.; Li, Y.G.; Ruan, K.P.; Zhang, S.M.; Zhang, J.L.; Kong, J.; Gu, J.W. Synchronously improved electromagnetic interference shielding and thermal conductivity for epoxy nanocomposites by constructing 3D copper nanowires/thermally annealed graphene aerogel framework. *Compos. - A: Appl. Sci. Manuf.* **2020**, *128*, 105670.
51. Zhan, Y.H.; Cheng, Y.; Yan, N.; Li, Y.C.; Meng, Y.Y.; Zhang, C.M.; Chen, Z.M.; Xia, H.S. Lightweight and self-healing carbon nanotube/acrylic copolymer foams: Toward the simultaneous enhancement of electromagnetic interference shielding and thermal insulation. *Chem. Eng. J.* **2021**, *417*, 129339.
52. Zhang, H.M.; Zhang, G.C.; Gao, Q.; Tang, M.; Ma, Z.L.; Qin, J.B.; Wang, M.Y.; Kim, J.K. Multifunctional microcellular PVDF/Ni-chains composite foams with enhanced electromagnetic interference shielding and superior thermal insulation performance. *Chem. Eng. J.* **2020**, *379*, 122304.
53. Zhou, B.; Li, Q.T.; Xu, P.H.; Feng, Y.Z.; Ma, J.M.; Liu, C.T.; Shen, C.Y. An asymmetric sandwich structural cellulose-based film with self-supported MXene and AgNW layers for flexible electromagnetic interference shielding and thermal management. *Nanoscale* **2021**, *13*, 2378–2388.
54. Zong, Z.; Ren, F.; Guo, Z.Z.; Lu, Z.X.; Jin, Y.L.; Zhao, Y.C.; Ren, P.G. Dual-functional carbonized loofah@GNSs-CNTs reinforced by cyanate ester composite with highly efficient electromagnetic interference shielding and thermal management. *Compos. B*

Eng. **2021,** *223*, 109132.
55. Pawar, S.P.; Stephen, S.; Bose, S.; Mittal, V. Tailored electrical conductivity, electromagnetic shielding and thermal transport in polymeric blends with graphene sheets decorated with nickel nanoparticles. *Phys. Chem. Chem. Phys*. **2015,** *17*, 14922–14930.
56. Yuan, Y.; Liu, L.Y.; Yang, M.L.; Zhang, T.L.; Xu, F.; Lin, Z.S.; Ding, Y.J.; Wang, C.H.; Li, J.J.; Yin, W.L.; et al. Lightweight, thermally insulating and stiff carbon honeycomb-induced graphene composite foams with a horizontal laminated structure for electromagnetic interference shielding. *Carbon* **2017**, *123*, 223–232.
57. Guo, Y.L.; Zhang, R.Z.; Wu, K.; Chen, F.; Fu, Q. Preparation of Nylon MXD6/EG/CNTs Ternary Composites with Excellent Thermal Conductivity and Electromagnetic Interference Shielding Effectiveness. *Chin. J. Polym. Sci.* **2017,** *35*, 1497–1507.
58. Yuan, Y.; Sun, X.X.; Yang, M.L.; Xu, F.; Lin, Z.S.; Zhao, X.; Ding, Y.J.; Li, J.J.; Yin, W.L.; Peng, Q.Y.; et al. Stiff, Thermally Stable and Highly Anisotropic Wood-Derived Carbon Composite Monoliths for Electromagnetic Interference Shielding. *ACS Appl. Mater. Interfaces* **2017,** *9*, 21371–21381.
59. Li, Y.; Xu, F.; Lin, Z.S.; Sun, X.X.; Peng, Q.Y.; Yuan, Y.; Wang, S.S.; Yang, Z.Y.; He, X.D.; Li, Y.B. Electrically and thermally conductive underwater acoustically absorptive graphene/rubber nanocomposites for multifunctional applications. *Nanoscale* **2017,** *9*, 14476–14485.
60. Liu, P.S.; Qing, H.B.; Hou, H.L.; Wang, Y.Q.; Zhang, Y.L. EMI shielding and thermal conductivity of a high porosity reticular titanium foam. *Mater. Des*. **2016**, *92*, 823–828.
61. Zhao, B.; Zhao, C.X.; Li, R.S.; Hamidinejad, S.M.; Park, C.B. Flexible, Ultrathin, and High-Efficiency Electromagnetic Shielding Properties of Poly(Vinylidene Fluoride)/Carbon Composite Films. *ACS Appl. Mater. Interfaces* **2017,** *9*, 20873–20884.
